# Use of digital healthcare solutions for care delivery during a pandemic-chances and (cyber) risks referring to the example of the COVID-19 pandemic

**DOI:** 10.1007/s12553-021-00541-x

**Published:** 2021-04-15

**Authors:** Florian Klaus Kaiser, Marcus Wiens, Frank Schultmann

**Affiliations:** Institute for Industrial Production (IIP), Karlsruhe Institute of Technology, Karlsruhe, Germany

**Keywords:** Digital health, Pandemics, Cost–benefit tradeoffs, Cybersecurity, Process mapping

## Abstract

During pandemics, regular service provisioning processes in medical care may be disrupted. Digital health promises many opportunities for service provisioning during a pandemic. However, a broad penetration of medical processes with information technology also has drawbacks. Within this work, the authors use the COVID-19 pandemic to analyze the chances and the risks that may come with using digital health solutions for medical care during a pandemic. Therefore, a multi-methods approach is used. First we use a systematic literature review for reviewing the state of the art of digital health applications in healthcare. Furthermore, the usage of digital health applications is mapped to the different processes in care delivery. Here we provide an exemplary process model of oncological care delivery. The analysis shows that including digital health solutions may be helpful for care delivery in most processes of medical care provisioning. However, research on digital health solutions focuses strongly on some few processes and specific disciplines while other processes and medical disciplines are underrepresented in literature. Last, we highlight the necessity of a comprehensive risk-related debate around the effects that come with the use of digital healthcare solutions.

## Introduction

During the coronavirus disease (COVID-19) pandemic, digital solutions have proven to be able to strengthen our healthcare system in emergency situations [1]. However, the example of using tracing applications for gaining control over the spread of severe acute respiratory syndrome coronavirus 2 (SARS-CoV-2) also showed that severe threats and risks may come with the utilization of digital solutions. Therefore, it needs to be carefully accessed where (fully) automated healthcare services are appropriate and where wide automation might not be reasonable [2]. This is especially relevant considering the increased vulnerability of the digital healthcare sector to cyberattacks during the COVID-19 pandemic [3].

The objective of this work is to provide an overview of the chances and risks taking a process-oriented perspective based on a multi-methods approach. In doing so our work provides a process model taking oncological care delivery as example and highlighting bottlenecks in care delivery during the pandemic. In order to get as comprehensive as possible an overview of the current state of research as well as of the range of promising devices, we conducted a systematic literature review investigating the usage of digital health solutions during the COVID-19 pandemic. We map the applied technologies to the respective processes discuss the chances and risks of implementing digital health applications in care delivery. The work intends to forward the scientific discussion on the digitalization of healthcare systems and cost–benefit tradeoffs that need to be considered when implementing digital health technologies.

The article is organized as follows. Chapter 2 presents the theoretical background and related work focusing on SARS-CoV-2 and COVID-19, digital health, and healthcare processes. Thereafter, a model of oncological care delivery during the pandemic is developed. It shows the state of the art of care delivery and highlights the challenges and specifics of healthcare during these times (e.g. bottlenecks). Chapter 4 provides a systematic literature review focusing on the utilization of digital solutions during the COVID-19 pandemic and maps the usage of digital technologies to the processes of care delivery. Chapter 4 presents a cost benefit discussion on the usage of digital health systems giving a differentiated overview on the risks and the chances that come with a high dependency on digital technologies for healthcare delivery. Finally, chapter 5 concludes the work and derives some implications.

## Theoretical background and related work

### COVID-19 and SARS-CoV-2

SARS-CoV-2 belongs to the community acquired respiratory viruses (CARV), which can cause upper and lower respiratory tract infections [4]. The virus was discovered in 2019 and describes a RNA-beta-coronavirus. COVID-19, the disease caused by SARS-CoV-2, is linked to various forms. While SARS-CoV-2 can cause asymptomatic infections it could also lead to severe forms of COVID-19 including severe viral pneumonia, “massive alveolar damage and progressive respiratory failure” [5], as well as an acute respiratory distress syndrome which could even cause death [6]. Within the year 2020, COVID-19 reached a pandemic spread, challenging the health system of nations worldwide.

The containment of the virus and the control of the disease were very challenging as interventions to the pandemic spread of the virus comprised drastic measures like mobility restrictions, physical isolation and quarantine [6]. As drug development cycles were long, there was no cure to the virus for a long time. Thus, the treatment of COVID-19 was largely symptomatic.

### Healthcare processes, value and the healthcare value chain

Measuring the performance of any system needs to be based on a common goal of each actor within this system (objective function). For economic systems, i.e. markets and value chains, this common goal predominantly is value optimization [7]. The term “value” describes the utility of process outcomes for the customers. “Value should be the preeminent goal in the health care system, because it is what ultimately matters for customers (patients) and unites the interests of all system actors. If value improves, patients, payers, providers, and suppliers can all benefit while the economic sustainability of the health care system improves. Value encompasses many of the other goals already embraced in health care, such as quality, safety, patient centeredness, and cost containment, and integrates them” [7]. Measuring this value starts with measuring the outcomes of health care. Thereby healthcare processes take a prominent role in the causality chain, which predominantly comprises processes of medical intervention (e.g. diagnosing or patient treatment) as so called primary value processes but also organizational processes (e.g. disinfection and cleaning) as so called secondary value processes. It is therefore essential to control processes as the source of value generation. Discussions about how to improve healthcare value and about the influence of technologies on healthcare need both to be based on the process level. Hence, modelling healthcare processes is essential for elaborating on the effects on healthcare value and understanding healthcare value generation.

### Digital health

“Digital health comprises overlapping areas ranging from AI, the internet of things, electronic health, and telehealth to the analysis and use of big data” [2]. This technological (r)evolution of the healthcare branch is said to be able to “support the provision of effective and efficient health care services” [8] and to stabilize the costs of healthcare delivery [8]. Furthermore, the digitalization of health delivery could enable holistic, personalized and precision medicine even for hard-to-reach-populations. Besides many different technologies for the digitalization of medicine, wearable technologies and mobile health applications may play a special role, as the market for wearable (medical) devices is a booming market with the momentum to significantly change the healthcare system as we know it [8]. Especially these wearable devices “are already revolutionizing biomedicine through mobile and digital health by enabling continuous, longitudinal health monitoring outside of the clinic” [9]. Furthermore, they simplify the monitoring of non-bedridden patients within a clinic. The chances that can be realized through digital health and the widespread use of data driven medicine can be shown considering the role of exogenous data (environmental influences), which can be integrated in data driven medicine but are oftentimes not used in traditional service provisioning. Moreover, traditional service provisioning is largely based on clinical data. A key opportunity for using digital technologies could therefore be to enable the usage of all available data from various data sources (big data) for determining individual health conditions. These various forms of health data are nowadays generated at a massive scale and on different levels. However, the usage in todays healthcare systems is low [10], many systems are in a preliminary stage (development) and therefore do not have operational maturity [11]. Furthermore, broad penetration of healthcare delivery through medical devices raises severe concerns about patients safety, security and privacy [12]. The chances and threats trough implementation of digital technologies in healthcare delivery raises tensions between the aim for a high quality care, resource efficiency (price efficient care) and the safety, security and privacy of patients and their health care information [12].

#### Chances of digital health applications for service delivery

As mentioned, transforming healthcare through digital health applications promises immense chances for service provisioning during pandemics. McCall [13] and Luengo-Oroz et al. [14] focus on applications of AI to cope with the COVID-19 pandemic. McCall [13] builds on the experiences of the SARS epidemic in 2003 and compares the coping capabilities with those of SARS-CoV-2 in 2020. Within this work, McCall [13] focuses on the usage of AI to predict COVID-19 outbreaks and their location. Luengo-Oroz et al. [14] focus on global cooperation and data sharing as a special issue and a necessary prerequisite of using AI. However, they only show very briefly some chances arising from using AI for designing a more resilient healthcare system with respect to pandemics. Kapoor et al. [15] focuses on the question of „how digital solutions can impact healthcare during (the) (…) pandemic” [15]. Within their work, they focus on the chances arising from a usage of digital solutions for healthcare (such as possibilities of tracking, telehealth, diagnostic support & information dissemination).

#### Risks for medial service provisioning

In May 2017, the WannaCry ransomware attack shed light to the threats of medical care dependence on digital technologies. This cyberattack was the first of its kind that caused severe disruptions of medical care delivery by causing digital medical devices (including medical imaging devices) to become non-operational. As a consequence, many hospitals were forced to forward patients to other hospitals and withhold medical services as well as to divert ambulance routes [16]. The wide usage of information technology in the healthcare sector brings numerous security vulnerabilities that could be exploited by attackers [16].

However, since 2017, driven by the potentials of using digital solutions for healthcare for medical care delivery, the digitalization of the healthcare branch increased. Consequently, medical care provision is facing new threats related to the increased penetration with information technology. These include the increased vulnerability (increased attack surface due to the medical internet of things; increasing inter-connections across hospitals and intra-connections within hospitals), increased exposure to potential attackers (increasing attractivity of attacking healthcare providers; increasing interest in attacking the healthcare branch as a valuable target, e.g. ransomware attack on devices care delivery is highly dependent on) as well as increased impact (increasing effects of cyberattacks as there is a high dependence on information technology in the modern healthcare branch).

#### Cost benefit tradeoffs

Keesara et al. [17] set the focus of their study on the comparison of the traditional analogous healthcare system and the benefits digital solutions for healthcare may bring to revolutionize this system. Furthermore, they shed light on the necessity to adapt the healthcare system to the needs of our todays digitalized, technologized and globalized world. Furthermore, they highlight barriers for the adoption of digital technologies (e.g. legal restrictions). Webster [8] compared selected experiences of clinicians with healthcare systems around the world. The work showed how different healthcare systems leveraged the power of information technology to deliver healthcare in the best possible quality during the COVID-19 pandemic. Although COVID-19 pushed digitalization forward, Webster [8] questioned if the penetration with information technology is sustainable or just transient for the period of the pandemic. Furthermore, their work showed the need for increasing digitalization for coping with the constraints and restrictive circumstances during the COVID-19 pandemic worldwide. However, their work strictly focuses on privacy risks regarding a potential digitalization of health care services lacking in a broader perspective on the risks that come with an increasing dependence on digital technologies such as security and safety risks and the wider effects of non-privacy preserving or insecure technology on public trust and the risks on healthcare technology adoption.

Our work aims to tackle this research gap and to forward scientific discussion on the question of where to reasonably apply digital technologies by giving a comprehensive overview about the chances and risks that are associated.

## Healthcare during the pandemic

### Effects of COVID-19 on healthcare

During the COVID-19 pandemic, an overriding priority on diminishing the spread of COVID-19 was observed for all healthcare systems worldwide. However, the impact of COVID-19 on the treatment of medical conditions, which are not linked to the disease must not be underestimated [18]. As a reaction to the COVID-19 pandemic, many hospitals “reduce or even cease many clinical services” [2]. Thereby two fundamental challenges arise concerning patient safety issues for patients with pre-disease. First, predisposed patients must leave their homes to visit the clinic and thereby possibly expose themselves to the infection [6]. Second, treatments themselves can predispose patients to more serious harmful effects of COVID-19 (e.g. cancer treatment) [6]. However, it is clear that postponing medical care cannot be done without time restrictions [2]. Postponements therefore need to prioritize the importance of medical care weighting the potential risks of pushing treatments forward with the risk of being infected by SARS-CoV-2 and the risks that may come with a postponement of medical interventions (e.g. risk for metastasis). The COVID-19 pandemic however revealed existing bottlenecks within many healthcare systems around the world with respect to critical care due to improper capacities (e.g. ventilators). Therefore, in many countries patients need to be postponed although they were infected by the virus and prioritizations of patients were undertaken. This stands in clear conflict with moral and ethical principles, in particular the Hippocratic Oath, which demands to provide the best medical care as possible to diminish harm and suffering. Therefore, the retention of capacities for urgent cases is prohibited as the denial of medical care would lead to a suffering from deprivation. Thus, non-COVID-19 patients must not be discriminated [19].

Cancer care is one example, where postponing medical care might be critical as without treatment the risk for metastasis increase [20]. However, cancer treatments such as surgeries are oftentimes postponed and laboratory evaluations delayed [20] [18]. Furthermore, post-operative care and aftercare examinations were suspended endangering the efficiency of cancer treatments. Yet, this may lead to increasing rates of readmissions. Moreover, cancer diagnosis was affected by COVID-19 as physician consultations were oftentimes postponed and diagnostic evaluations were delayed due to resource restrictions [21] [18]. This might be critical to the individuals health condition as early detections are essential for cancer treatment and advanced forms of cancer are oftentimes less amenable to medical interventions [22]. Salako et al. [23] predict that with changed treatment procedures during the COVID-19 pandemic medical outcomes will worsen, leading to higher mortality rates due to improper treatment. However, oncological care outcomes must not be compromised while minimizing patients exposure to SARS-CoV-2 [24].

### Modelling oncological care processes

#### Methodology for modelling health care processes

Various methods for modeling processes exist. These include business process model and notation (BPMN) and Integration Definition (IDEF). IDEF is a set of process modelling techniques including six specifications (IDEF-0 – IDEF-5). The most commonly used form of IDEF modelling is IDEF-0 modelling, which is also used in this work. IDEF-0 was chosen because it allows an intuitive representation and a process oriented mapping of information technology along the healthcare value chain which is essential for discussing the potentials and risks of an increasing penetration with information technology and new technological innovations in healthcare for supporting our healthcare system during a pandemic.

**Fig. 1:**
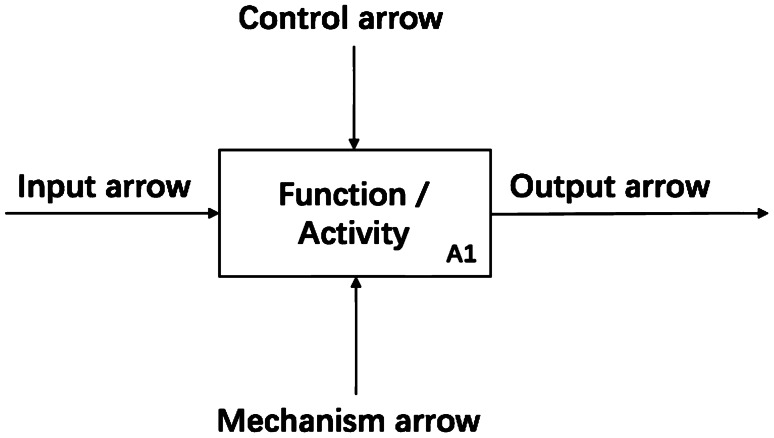
IDEF-0 model components

IDEF-0 models are represented as a graphic description with the format of boxes and arrows. The basic components of an IDEF-0 model are shown in Fig. 1. The boxes in the IDEF-0 model are used to represent functions and arrows depict constraints [25]. The function is responsible for the transformation of input material flows into outputs by using diverse resources under control constraints [26].There are four arrow classes including input, output, control and mechanism arrows. The activity transforms input data or objects into output material flows, which is represented by input and output arrows [25]. The control-arrow above the activity box represents the required conditions for the output production such as rules, regulations, policies, etc. [25]. The mechanism-arrow below the activity box represents tools, methods, and different resources for the activity execution [25].

#### Oncological care delivery model

**Fig. 2:**
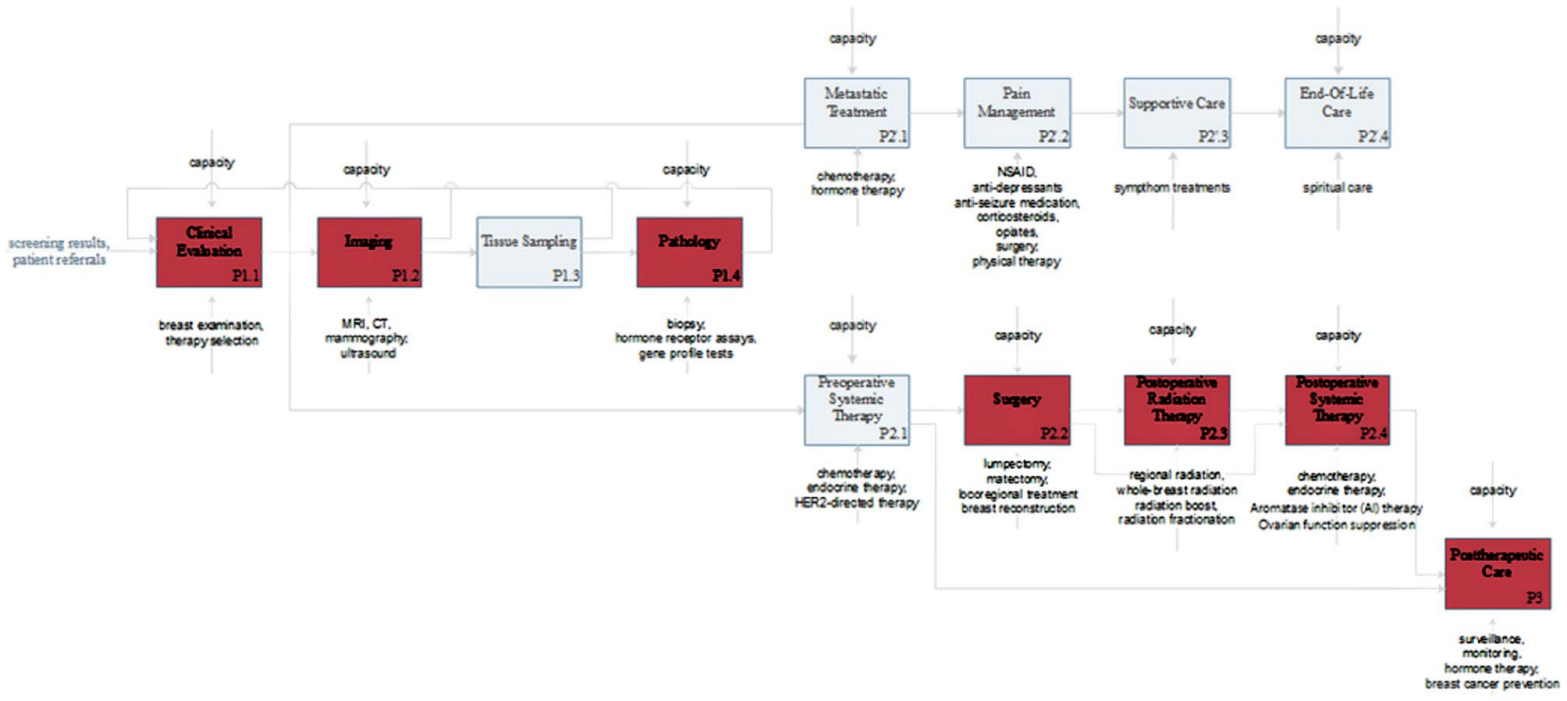
IDEF0 model of breast cancer healthcare

In the following, a model of breast cancer care will be presented. The example was chosen because oncology patient treatment procedures have been affected severely through COVID-19 as patients are a highly vulnerable group due to their immunocompromised health condition. The high vulnerability for SARS-CoV-2 is thus caused by cancer itself but also by the treatment of cancer relying heavily on the intake of immune system suppressing drugs [27]. Figure 2 presents a process model, which gives a basic understanding of medical care provision and the effects of the COVID-19 pandemic on care provision using the example of breast cancer care. The model is based on IDEF0. The processes P1.1—P1.4 describe the diagnostic phase while P2.1—P2.4 describe the treatment phase for metastatic cancer where palliative care must be chosen and P2.1 -P2.4 describes curative therapy for early, localized or operable breast cancer. P3 represents post-therapy care.

The first phase of clinical breast cancer care is the diagnosis. Clinicians evaluate the health condition of the patient confirming a breast anomaly [28]. The confirmation of a breast cancer diagnosis requires an evaluation of the stage of the disease, the selection of a therapy, and subsequent tests including imaging (mammography, ultrasound and MRI) and a biopsy [29]. If the cancer can be treated, there are some standard treatment options including the surgery options of a breast-conserving surgery (lumpectomy) and a modified radical mastectomy [29]. Systemic therapies include e.g. adjuvant therapies like a chemotherapy [29]. However, if the cancer cannot be treated, clinician health care processes are limited to palliative care of the patient such as pain management [28]. Post-therapy considerations include surveillance and hormone replacement therapy [29].

As identified in the literature, there are several bottlenecks in health care provision for cancer patients which relate to surgeries, laboratory evaluations (esp. pathology), diagnostic processes and postoperative care [20] [18]. These main bottlenecks (processes) are represented within the breast cancer treatment process model (Fig. 2) in red.

Thereby it is an essential duty of modern healthcare systems during a pandemic to minimize deprivation costs stemming from a lack of medical care. However, the costs of suffering from a lack of medical care need to be weighed against the risks of potential infections when deciding on whether to treat a patient or postpone medical interventions.

## Literature review on existing technologies to tackle the COVID-19 pandemic

### Methodology

As it was stated by Ienca and Vayena [30], SARS-CoV-2 emerged in a very digitized world, which bears many possibilities to contain and cure the COVID-19 disease by leveraging the power of data and digital health. We conducted a systematic literature review to investigate implemented technologies and potentially useful technologies to cope with the effects of the COVID-19 pandemic.

For systematically assessing literature regarding the use of digital technologies for healthcare delivery during the COVID-19 pandemic we used the literature database Scopus. We defined the inclusion criteria presented in Table 1. For setting the thematic focus on digital health solutions are extracted keywords from the work of Ahmadvand et al. [31] elaborating on keywords linked to the term digital health (most frequently linked terms include inter alia "mobile health", "mhealth", "health IT", "telehealth", and "telemedicine"). The literature search was conducted in July 2020.

**Table 1:** Literature search

Criteria	Inclusion
Thematic focus on the COVID-19-pandemic	Title, abstract or keywords include the terms COVID*, corona or SARS-CoV-2
Thematic focus on digital health solutions	Title, abstract or keywords include the terms digital health, mobile health, mhealth, health IT, health information technology, wearable devices, telehealth, telemedicine or personalized medicine
Publications are recently published and peer reviewed	Publication stage is final, publications are from peer reviewed journals and the year of publication is 2020
Language of publication is English	Language of publication is English
Resulting search term	TITLE-ABS-KEY(("COVID*" OR "corona*" OR "SARS-CoV-2") AND ("digital health" OR "mobile health" OR"mhealth" OR "health IT" OR "health information technology" OR "wearable devices" OR "telehealth" OR "telemedicine" OR "personalized medicine")) AND ( LIMIT-TO ( PUBSTAGE,"final")) AND ( LIMIT-TO ( PUBYEAR,2020)) AND ( LIMIT-TO ( DOCTYPE,"ar")) AND ( LIMIT-TO ( LANGUAGE,"English"))

**Fig. 3:**
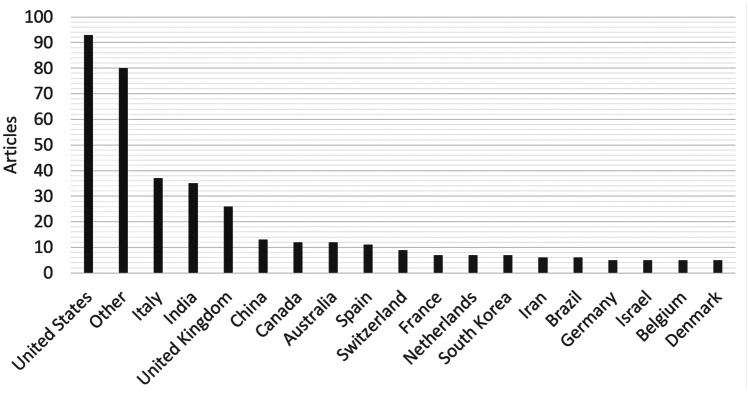
Articles by country

The resulting literature search included 346 articles, which were further investigated. 96 articles need to be sorted out because they were not of interest for this study. Therefore, the resulting literature review consists of 250 articles. Figure 3 presents the articles by country. Within this presentation, we used a limit of 5 article contributions per country for the sake of clarity, if a country has less article contributions we summarized these within the category “Other”. It can be seen that especially countries heavily influenced by COVID-19 elaborated on possible use cases of digital health for medical service delivery.

We categorized digital health applications and medical devices deductively within the categorization of medical processes and organizational processes. Furthermore, we clustered medical processes in a more detailed way within the categorization provided by Naudé [11] (“tracking and prediction”, “diagnosis and prognosis”, “treatments and vaccines”, and “social control”).

## Results

### General overview of the application of digital health applications

The systematic literature review shows that especially during the COVID-19 pandemic digital health technologies (e.g. tele-diagnosis or telecare) helped substantially to make the healthcare system more resilient and efficient by providing healthcare solutions to a wide array of value processes. Digital technologies may for example “enable that patients have access to physicians (…) while remaining safely at home” [32] and without exposing themselves to the risk of an infection. For some vulnerable groups, telehealth solutions can even be the only way to access healthcare. Telemedicine may therefore help to ensure that everybody gets access to medical care [33]. However, besides these aspects where digital health technologies are considered helpful or are used in primary value processes (e.g. diagnosis and treatment) [33] [34], technological advances are also used in supportive processes (so called secondary value processes) [35] [34] [36]. However, there is a strong focus of research on the effects and usage of digital technologies for primary value processes. Furthermore, tracking and prediction as well as social control are further processes associated with healthcare delivery that can be supported by digital health [37]. Hence, digital health applications provide solutions or are as least discussed to be able to deliver solutions for all processes within medical service delivery including medical processes as well as organizational processes [36]. The frequency of keywords can here be seen as a proxy of the expected usefulness, since it can be assumed that only those technologies with a high number are included in the scientific discourse, which promise a significant benefit.

In the countries of India and China robots are for example used to assist frontline healthcare workers (especially nurses) by delivering food and sanitizing [38]. In situations like the COVID-19 pandemic, digital health devices deliver valuable data for public decision-making. However, it does also deliver valuable data for clinical decision-making and can aid in healthcare processes like diagnosing, managing and treating as well as in prediction of the course disease. Thereby also fitness trackers may deliver valuable insights, which can be used to improve care delivery and enable (even personalized) health care interventions.

Furthermore, IoT devices can be used for medical imaging [35]. Robotic ultrasound equipment can be used for remote high resolution diagnostic imaging [39]. Digital health solutions can be used for evaluation and suggestion of therapies [40]. This can be based on predictions regarding clinical outcomes [38]. Furthermore, robots are useful for assistance in surgeries and can be combined with information and telecommunication technologies for enabling tele-surgeries [40]. Additionally, robots can assist in treatments of patients, perform online medical examinations and perform real time monitoring [35]. However, the most common use case is the enablement of tele-medical communication and virtual patient visits. AI and information technology can be used to support diagnosis (e.g. screening) and treatment (individual ventilator settings) [38]. Furthermore, secondary value processes like drug development and other services (e.g. providing food taking care, sanitizing, and surveillance) can be supported by digital health solutions [38]. Furthermore, virtual reality can be used for training purposes [35]. Besides medical processes in the narrow sense, digital health solutions can be used for tracking and tracing health conditions within a society and controlling the compliance with prescribed procedures (e.g. wearing masks). Based on the big amount of data valuable predictions can be generated by leveraging the power of AI (forecasting, classification, identification of health conditions like COVID-19 and illnesses that come with similar symptoms but are not linked to COVID-19, alerts, and trace infection hubs) [35] [38].

**Fig. 4:**
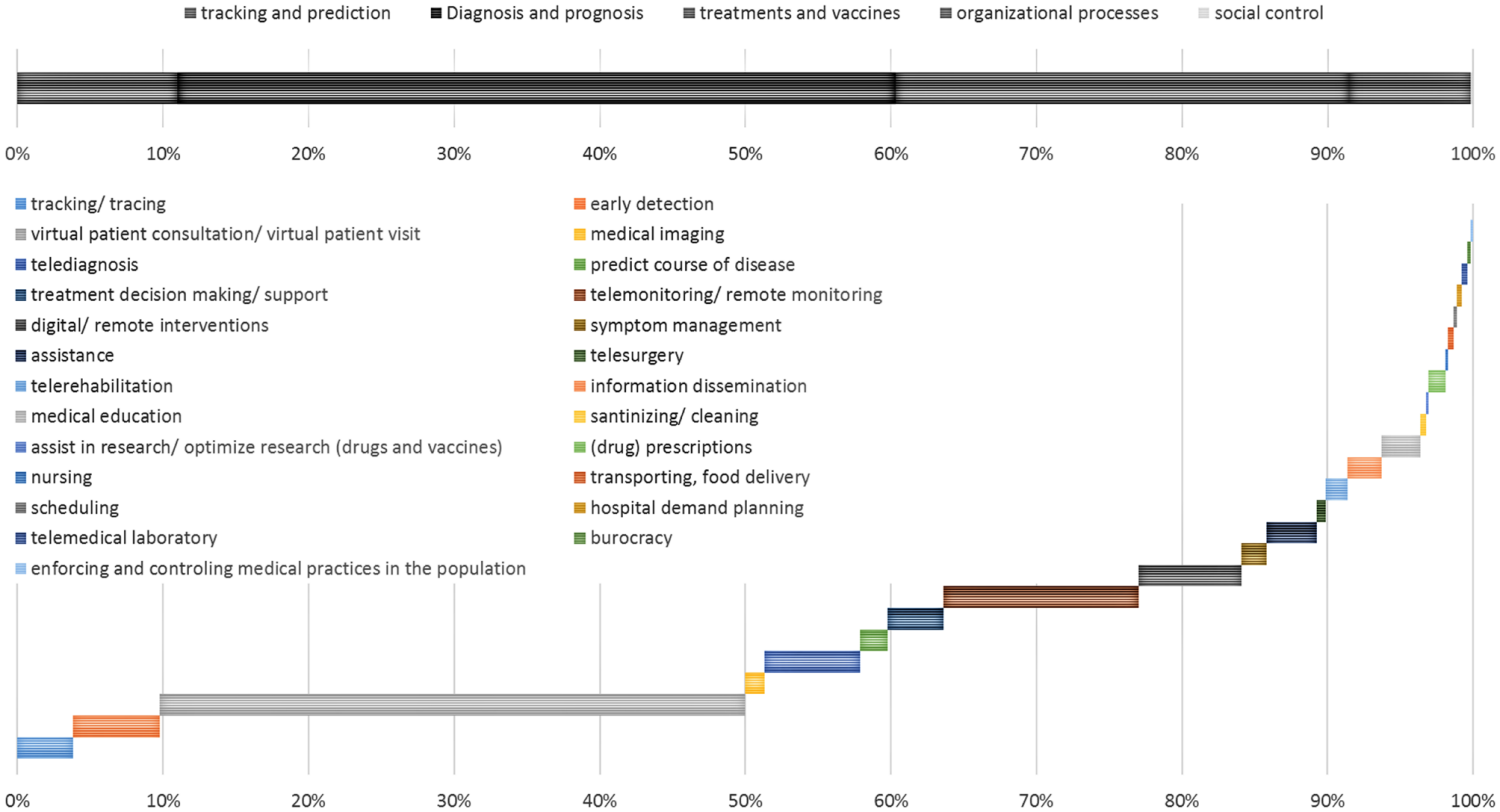
Shares of articles proposing the use of digital health in the respective medical care process

Figure 4 visualizes the shares of articles elaborating on using digital health applications for different processes of medical care and therefore the expected usefulness of including means of digital health to those processes. The upper part of the figure shows major processes of medical care systems while the lower part visualizes the usage of digital health in specific subprocesses. The literature review reveals a focus of scientific research on the support of medical processes (diagnosis and prognosis and treatments and vaccines). Within medical processes, the main potential of including digital health applications to medical care during pandemics is seen in enabling patient consultations by virtual means followed by possibilities for remote monitoring and remote interventions.

**Fig. 5:**
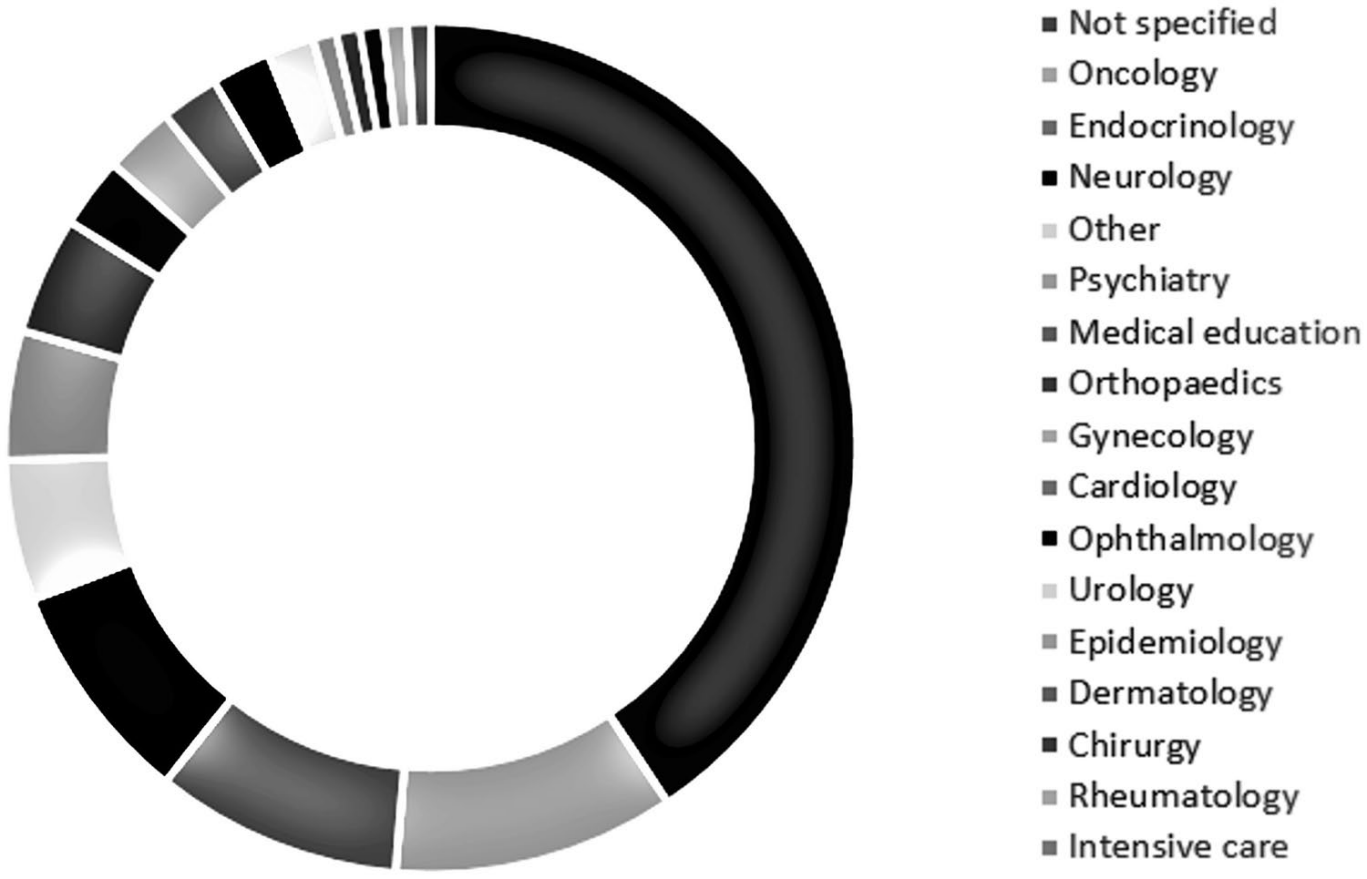
Distribution by medical department

Figure 5 gives an overview of the usage of digital health devices in different medical departments. It needs to be emphasized that digital health solutions are used in some medical disciplines since many years (e.g. oncology, endocrinology esp. diabetes care and psychiatry) [41]. However, the adoption of digital technologies in other specialties is much weaker. Furthermore, a large amount of articles dealt with the usage of digital health applications during the COVID-19 pandemic in general (“not specified”).

### Applications in oncological care

**Fig. 6:**
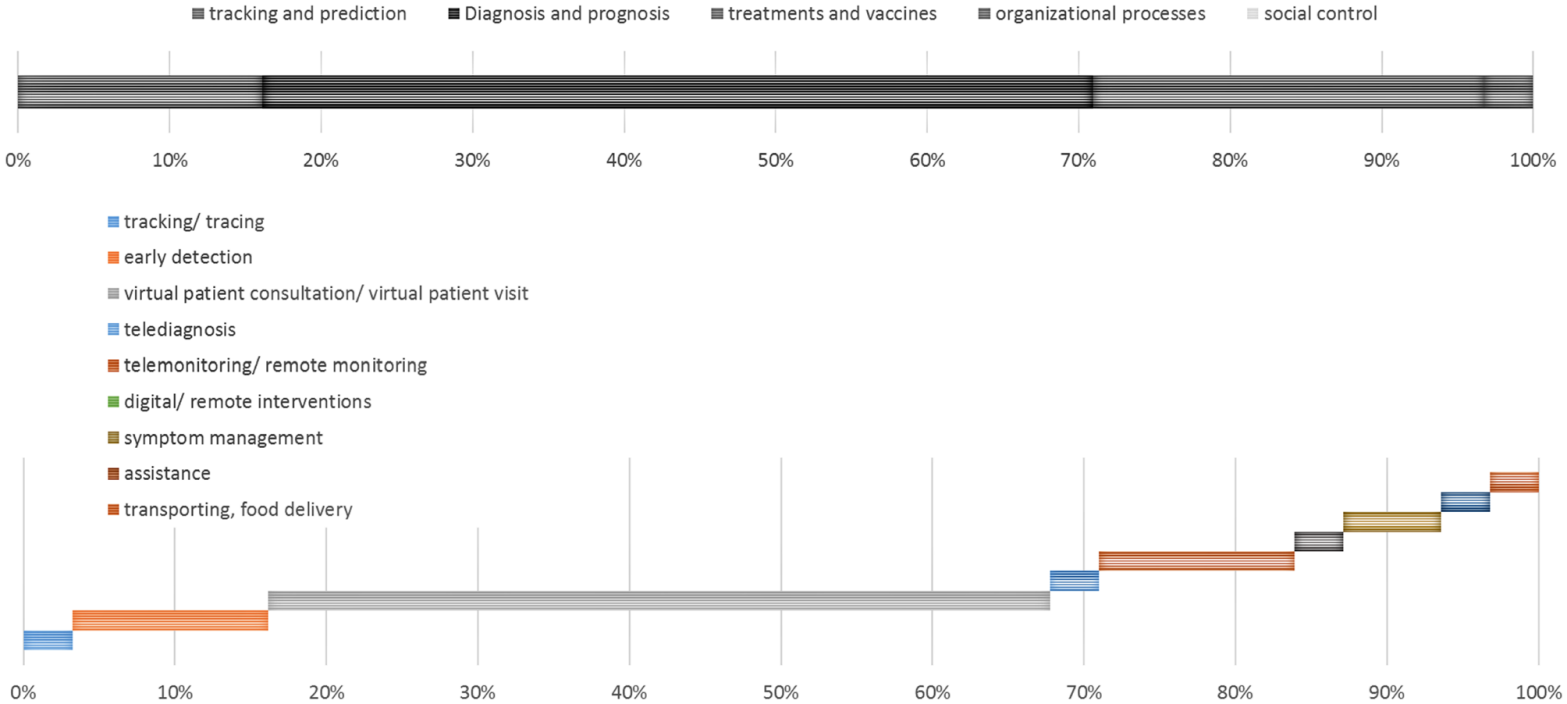
Shares of articles dealing with different processes of oncological care

**Fig. 7:**
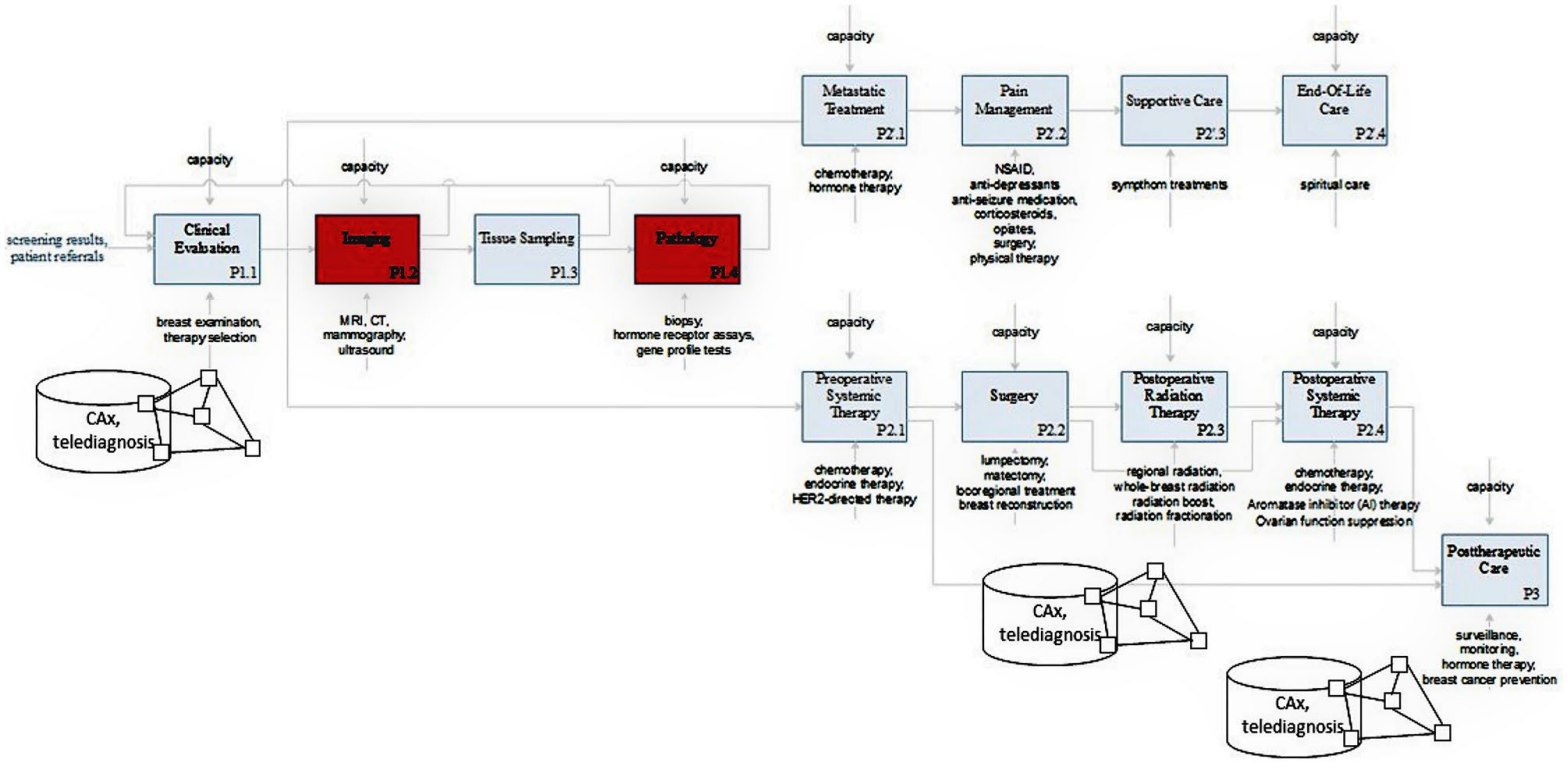
Digital solutions for healthcare in breast cancer healthcare

For mapping the digital health solutions to the processes of breast cancer care, we restricted our review only on those 24 articles dealing with oncological care. Thereby secondary value processes were not in the focus of the publications (see Figs. 6 and 7). Instead, they focus merely on the specifics of treatment of oncological diseases and therefore do not include general usage of digital solutions for increasing the efficiency of healthcare delivery. Furthermore, it catches the eye that virtual patient visits are most frequently considered as a possible use case of digital technologies in oncological care (like it was also focused most frequently for all medical departments). Additionally, the possibilities of using digital health applications for diagnosing are discussed frequently (usage of digital health applications for “early detection”, and “telediagnosis”). Last, “telemonitoring” and the usage of digital health for enabling interventions is considered to be beneficial. However, the potential for enabling interventions can be estimated to be lower in oncology compared to the overall use in medicine.

### Mapping to the IDEF0 model

Digital technologies may help to overcome at least some challenges in cancer care delivery so that in some cases cancer treatment could be done virtually [42]. An example for the possibilities is the usage of AI to enable the evaluation of big data generated through digital technology, which enabled wide screening of mammograms while overcoming high false positive rates in human expert predictions [22]. This makes it possible to overcome some bottlenecks in diagnosing cancer diseases and can therefore diminish the burden to clinicians. Thus, the caseload of a clinician could be increased in order to cope with the high number of patients during a pandemic. However, there will remain some bottlenecks in healthcare (see red processes in Fig. 7) where information technology currently does not provide significant help including imaging and pathology processes. The reason is that digital solutions for healthcare are strong in e.g. recognizing patterns in images and derive treatments (decision support) but do not provide solutions for the problem of shortages in information technology that generates these images (e.g. shortages in computer tomography (CT) machinery). For specific applications, medical technology has higher requirements than widely adopted, “classic” smart technologies. For example, although smartphone cameras could theoretically be used for medical imaging [34], these technologies are not able to take medical useful images in the case of breast cancer. Therefore, these technologies are not suitable for medical imaging. Furthermore, digital solutions for healthcare can help to decrease the burden in treating patients (e.g. decision support). The technologies, which are especially useful for medical care of breast cancer patients include telehealth services (teleconsultations, tele-diagnosis, and tele-monitoring), which is also useful for decreasing crowded waiting rooms and hence the risk of an SARS-CoV-2 infection, and monitoring of health conditions leveraging the power of wearables and other smart devices (using health applications).

## Discussion

### Chances of introducing digital health to cope with pandemics

Digital technologies can be used to support healthcare provision in many ways [43]. Digital solutions for healthcare can be used in diagnosis (e.g. computer aided detection (CAD), tele-diagnosis, AI assisted decision making, screening; e.g. breast cancer screening [22]), treatment (e.g. decision support based on AI, symptom management and monitoring of health conditions, tele-surgery) and aftercare (e.g. monitoring of health conditions) to overcome decision errors (i.e. high false positive rates in breast cancer diagnosis undertaken by healthcare professionals) [43]. Furthermore, digital solutions for healthcare may be a means to enable access to medical care for hard to reach populations through telehealth and the empowerment of patients in care provision. Furthermore, digital health including AI can be used to detect diseases, for predicting disease progression as well as enabling broad screenings among large parts of the society [2] [13] [14].

Furthermore, for public information dissemination also the internet of things (IoT) can be used [2]. AI and Deep Learning can be used to fight disinformation [14]. Besides, these technologies can be used to create human like chat bots, which can deliver information in a more accessible way. Using all available types of data (e.g. genome data, protein structure, clinical data, medical image data, case statistics, epidemiological data, mobility statistics, and scientific research outcomes) is challenging. Therefore, big data analytics is necessary to leverage all potentials of using medical data.

Furthermore, the inclusion of such technologies may provide a benefit to patients and empowers their role in care provision (e.g. self-monitoring) [43]. Digital solutions for healthcare may therefore leverage the power of medical data processing and analysis, health data mining as well as health data modelling for computer-aided healthcare (such as computer-aided diagnosis and computer-assisted surgery). Using digital technologies for healthcare may furthermore increase the caseloads of physicians without increasing the workload as physicians can focus themselves on their task without being distracted by routine tasks that could easily be done by digital health solutions [32] [35]. Furthermore, travel times can be minimized and thus further diminish the workload of physicians [35] and speed up some processes. This is because the assistance through digital health applications may simplify (e.g. through the takeover of administrative issues) or even take over significant parts of the provision of medical care. Additionally, patient consultations could happen largely virtually [32]. Digital solutions for healthcare like online cognitive behavioral therapies or counselling services pose great chances for care delivery and are widely used for (especially mental) care provision [44]. Therefore, information technology may contribute to holistic healthcare provision and precision medicine. Additionally, digitalization of healthcare can be seen as a means for making medical service provisioning more (at least socially and economically) sustainable even in extreme situations (e.g. during pandemics).

Furthermore, digital health could be and is already used in research for identifying applications of known drugs to the cure of new diseases (e.g. COVID-19) [13] or accelerating the development of innovative digital health care applications [14]. However, there is a need of high quality input data [13]. Application areas of digital solutions for healthcare in research are for example AI assisted drug discovery and discovery of vaccines [33].

These chances of including digital solutions for healthcare may improve patient care. However, the increasing usage of mobile applications like health apps or wearables may further increase the potential of digital solutions for healthcare as data availability increases with the increasing share of mobile application usage. Although there are big chances of using digital solutions for healthcare, so far there is only limited usage in general. Furthermore, usage of such technologies is oftentimes limited to specific phases of care provision and predominantly used for curing specific diseases (e.g. diabetes, psychological disorders) [43]. However, “there should be a higher degree of pervasiveness at all stages and in all health care delivery activities” [43].

### Risks of digital health devices

Besides the immense opportunities inherent in a wide usage of digital solutions for healthcare, the strong dependence on digital health infrastructure increases the vulnerability of healthcare service providers to cyberattacks. Especially during a pandemic where work- and caseload for physicians is very high, these cyberattacks may have an immense impact on the proper functioning of healthcare provision. An outage of digital solutions could then lead in the worst case to a complete quiescence of operations, which would have severe effects on health care value. Consequently, the impact of a cyberattack may get more severe if service provisioning is highly dependent on digital solutions for healthcare. Therefore, attacking health care providers may get more attractive [45].

As a consequence, privacy and security are essential for the acceptance of digital health but cyber threats represent a significant barrier for the implementation of digital solutions in healthcare [14]. For example, tracing and tracking apps (like they were in use during the COVID-19 pandemic) may represent a significant threat to health data privacy and also to human rights in general. These tracing and tracking applications rely on the assumption that gaining “control of the pandemic requires (gaining) control of people” [1]. However, these applications may pose severe restrictions of basic human rights as authorities widely collect more data than an individual may agree on when deciding rationally [11]. Especially past crises should be alarming when deciding about solutions compromising human rights as they proved that regaining these rights can be hard [1] [46]. Thus, it is essential for digital solutions for healthcare that they respect basic human rights, also during pandemics [46].

However, as there may be a high necessity to develop these innovative digital health care solutions (e.g. during a pandemic) there is a shortage of time for development. The immense time restrictions of developing solutions e.g. for the COVID-19 crisis poses the necessity of prioritizing [14]. Thereby security and privacy must not be neglected (as discussed). The actionist development of digital solutions for healthcare and frugal innovations during the COVID-19 crisis can be seen as an example. Many digital solutions for healthcare were introduced during that time [46]. However, it was shown that the majority of applications to cope with COVID-19 compromised essential human rights (especially privacy rights) [46]. Therefore, there are severe concerns about the threats that are posed by digital solutions for healthcare (that are not constructed with a focus on security and privacy) to human rights [14]. “Useful as these are, the fear is that once the outbreak is over, that erosion of data privacy would not be rolled back and that governments would continue to use their improved ability to survey their populations” [11]. These concerns may limit the acceptance of digital health care solutions by society in a long term and may hamper trust in medical devices and authorities [11].”Data breaches, insufficient or ineffective de-identification and biases in datasets can (consistently) become major causes of distrust in public-health services “ [30]. Therefore, privacy and security is essential for digital health solution providers to be able to build trust in the usage of digital health solutions and use the chances that are inherent to the use of these technologies [45].

The COVID-19 pandemic boosted adoption of digitalized healthcare. However, there was no wide readiness within the healthcare systems in many countries. For example, there were oftentimes lacks in necessary hardware and technical knowhow to enable digital care delivery [32] [47]. Furthermore, technological solutions for healthcare pose their own specifics on medical staff such as knowledge about how to work with these technological solutions properly. The questionable readiness of many healthcare systems to adopt and integrate these technologies into their daily operations could thus have led to an increased susceptibility for cyber risks and to the fact that the chances were not entirely realized. Hence, it could not be observed that digitalized healthcare systems were able to cope with COVID-19 significantly better. Additionally, the COVID-19 crisis showed that cyberrisks need to be taken seriously as the dependence on digital technology increased when traditional means of delivering healthcare were limited severely. This can also be seen in the increasing number of cyberattacks during the COVID-19 pandemic [3]. It is therefore essential to design digital healthcare systems with a strong focus on security and privacy. Thus, the engineering of secure systems for healthcare services is imperative.

## Conclusion and implications

The COVID‐19 pandemic could alter sustainably medical practice [48]. Hence, it is important to jointly discuss the chances and risks that come with an increasing penetration with digital health solutions. With the intent to forward scientific discussion on the cost–benefit tradeoffs on digital health devices, this article gives an overview of the influence of digital solutions on the healthcare sector by relying on a multi methods approach. Based on recent research presented in literature, the chances as well as the risks arising from an inclusion of digital solutions for healthcare on medical care provision are presented with a special focus on the resilience of healthcare systems during a pandemic. In particular, the work presents a process model of care delivery and maps technological usage to the affected processes.

Pandemics may disrupt traditional (non-digital technology based) care delivery processes (e.g. during the COVID-19 pandemic), the importance of including digital solutions for healthcare rises. Thus, increasing the wide adoption of digital technologies in healthcare could decrease the burden to clinicians in pandemics and hence increase the resiliency of our healthcare systems. As digitalization of many areas in medical service provisioning is a fact, choosing the right digitalization strategy gains in importance for modern healthcare systems around the globe. The adoption and usage of digital technologies in healthcare is thereby mainly driven by the tradeoff between healthcare costs and quality of services (benefits). While the discussion on healthcare digitalization oftentimes focuses on the chances and direct monetary costs, risk are overlooked or considered insufficiently. Consistently, many risks that come with the adoption of digital health technologies are only discussed and even understood inadequately.

This work aims at highlighting the needs and sharpening the awareness that decisions on the adoption of digital health systems should be based on a comprehensive/ holistic assessment of both costs and risks. We therefore give an overview over the chances and risks arising from the adoption of digital technologies with a special focus on healthcare during pandemics. We show that there are great opportunities for a long number of processes including organizational processes (secondary value processes) as well as primary processes of medical service provisioning (including diagnosis and treatment). However, it need to be ensured that patient safety and data privacy is not jeopardized trough cyberattacks. Therefore, engineering secure systems and guaranteeing security, safety and privacy when using digital solutions for healthcare is a necessary prerequisite for being able to leverage on the power of an inclusion of digital technologies in healthcare. We furthermore take a multi perspective view on the chances and risks including social, economic and medical points of view. Moreover, we include a wide array of different risks (inter alia privacy and security risks) to our analysis.

The COVID-19 pandemic should be used as a natural experiment to analyze the effects of shifting processes to digital areas. Hence, in aftermath of the pandemic effects of improper service provisioning should be evaluated. Furthermore, the effects of digital health technologies should be considered. Thereby a focus should not only be set on whether digitalization increased the efficiency and effectiveness of service provisioning but also on cyber risks and the susceptibility of healthcare provisioning systems.
